# Governance in the era of Blockchain technology in Qatar: a roadmap and a manual for Trade Finance

**DOI:** 10.1057/s41261-021-00165-1

**Published:** 2021-09-03

**Authors:** Imad Antoine Ibrahim, Jon Truby

**Affiliations:** Center for Law and Development, College of Law, Qatar University, New Faculty Bldg. I03. – 618, Doha, Qatar

**Keywords:** Blockchain, Trade Finance, Qatar, Financial technology, International regulations and standards, National regulations, Sandbox regulations

## Abstract

Nations worldwide have sought to capitalize on the benefits of distributed ledger technology (DLT) including Blockchain, but struggled to strike a balance between encouraging investment and innovation in the technology while addressing the challenges and uncertainties through regulation. Through its FinTech (Financial Technology) Strategy, Qatar has sought to embrace DLT, but its regulatory approach also remains cautious. Trade Finance is an ideal business process to be disrupted through the benefits of DLT and especially Blockchain technology, since its processes remain antiquated, inefficient and lack digitization. Blockchain as a form of DLT particularly offers the Trade Finance process not only more rapid, secure, cost-effective and efficient procedures, but importantly completely assures trust between importers and exporters and removes the requirement to place such trust in third-party intermediaries. Qatar can reap considerable economic benefits through the enhancement of its Trade Finance regulations enabling the adoption of such Blockchain technology. As such, the authors propose a roadmap and manual for the governance of the Trade Finance Blockchain ecosystem in Qatar. The authors propose multi-layered governance approach to the regulation of Blockchain in Qatar by (1) embracing international regulations and standards; (2) replicating foreign regional and national rules that are appropriate and innovative; and (3) applying sandbox regulations to Blockchain products and services.

## Introduction

Having proven its capabilities and benefits, Blockchain [96, 178] as a form of DLT continues to be embraced across industries to disrupt and consequently improve business processes [20]. Nations and global organizations [53] study its applications and development, while various Blockchain industrial consortiums have been established to promote it and ensure its dissemination and usage. Some countries such as the USA have allowed for regulatory flexibility to encourage innovation in this field, whereas other nations like China have adopted a more cautious attitude [72].

Regulatory frameworks surrounding the use of DLT lack clarity given the nascent stage of the technology, while stakeholders press for the adoption of rules that create a stable playing field [22]. DLT has the potential to change business processes in innumerable sectors that rely on lengthy paperwork procedures, while simultaneously increasing security and transparency and reducing costs. Trade Finance depends upon these characteristics and as such is a sector that can considerably benefit from disruptive technology offered by DLT including Blockchain, as demonstrated by major banks including Barclays and HSBC [145, 155].

Qatar is one of the countries seeking to pave the road globally in FinTech. Qatar’s policy-makers adopted a cautious approach addressing DLT’s risks by banning specific activities depending on DLT, while acknowledging its potential benefits by including it in its FinTech strategy [3, 28, 73, 133]. This article evaluates how Qatar can regulate the use of DLT in Trade Finance to capitalize on its benefits while avoiding its risks. The article will highlight that Qatar should adopt a multi-layered governance approach to the regulation of DLT in Trade Finance by (1) embracing international regulations and standards; (2) replicating foreign national rules that are appropriate and innovative; and (3) applying sandbox regulations to DLT products and services.

The article will first provide an overview of the benefits and drawbacks of such application of DLT from a legal and technical perspective. The focus will be on Blockchain given its prevalent usage in Trade Finance compared with other types of DLT, though policy recommendations provided will cover DLT generally. The authors will then examine Qatar’s cautious attitude in its governance of DLT, identifying the potential benefits and recommending a progressive measured roadmap for Qatar’s DLT Trade Finance ecosystem, based on a multi-layered governance approach that includes the three legal tools mentioned above. Through this multi-layered governance approach, the authors are looking to have a unified strategy with other countries to make cross-border trade in finance using Blockchain easier and more efficient.

## Benefits of a switch to DLT Trade Finance

A number of problematic areas in the existing Trade Finance sector can be identified as follows:

1. A global gap in Trade Finance exists as a result of rejection of the amount of Trade Finance requested by importers and exporters;
2. The processes of Trade Finance are only partially digital as data are entered “manually without automated cross-checking, with parties submitting financial data on a spreadsheet or via printed and scanned documents”;
3. There is a lack of one single platform connecting all the actors of Trade Finance (buyer, seller, their banks, insurance providers, logistics companies, etc.) forcing them to connect via multiple platforms; and
4. Trade Finance operations are still risky due to the lack of connectivity requiring the use of various systems, delaying the effective digitization of the sector and time wasting particularly as errors occur often [131].

To be efficient, a “bank’s Trade Finance offerings must be agile, low cost, and valued by customers”. DLT can help the Trade Finance sector achieve these objectives [27]. The idea of implementing various innovative solutions in Trade Finance to address existing shortcomings is not new as scholars and experts have been attempting to solve the existing challenges before the emergence of DLT, [16] and there are alternative possible disruptive technologies that will not be examined herein [27]. Even with Blockchain as a form of DLT at a relatively new phase of its development, it has demonstrated significant potential in Trade Finance [11].

While there are alternative types of DLT available, one of the more common DLT types to be deployed in Trade Finance is Blockchain, given its clear advantages as described as this focus point. The main objective from the use of Blockchain technology in Trade Finance is ensuring trust between importers and exporters particularly when it comes to payment [103]. Parties can perform an entire transaction without depending on a third-party intermediary such as banks to provide trust and security [8]. Trusted intermediaries are removed from the business process, which reduces security risk, removes the requirement for both parties to trust the intermediary and saves cost. The Blockchain process is both public and immutable, relying solely upon the contracting parties involved to enter their own data, so does not require parties to trust a third party.

The decentralization and accessibility to the network by all participants help “…in tracking bills, goods and payments leading to increase in speed and reduction of counter party risks” [71]. The decentralized nature of the technology “facilitates and expedites collaboration and activity between parties while reducing the data’s susceptibility to being hacked, lost, changed, or destroyed” [8]. This is possible since information is digitized where smart contracts are used to initiate commercial transactions automatically [155]. As a result of decentralization and replication, the participants can have a full audit trail that can be verified by all of parties because of the inbuilt cryptographic integrity checks, lowering the need for trust in central hubs. If some nodes fail or are disconnected, this does not affect the rest of the nodes since all participants have a copy of all the data, in contrast to traditional financial institutions where if one central institution is down, the entire system is interrupted. There is also the byzantine fault tolerance addressing nodes that malfunction or are functioning in a malicious manner. This may occur because of cyber-attacks, terrorist groups or gangs looking to steal money or corrupting and destroying the data [173].

The trust in transactions increases when using Blockchain as the changes made can be viewed by all the parties, while the transactions cannot be deleted from the ledger, thus enhancing transparency [8]. This leads to increased transparency especially as audit trails are created as transactions are recorded in order and goods are tracked throughout the “supply chain and relay information to and from the owner” [155]. The security is maintained as Blockchains cannot be changed unless the participants agree to do that where each party can see all the information related to the interconnected blocks from the past until now [8]. Complex cryptography is used to verify transactions, making sure that information is authentic and secure [155]. Costs are reduced due to the absence of a third party as the ledger is located across all of the network’s interconnected devices. Time is also reduced since there is no reliance on a third party, such as a bank, to verify the payment process [8].

Blockchain and other types of DLT are not “a solution in search of a problem” but solves a problem by removing the need to trust an intermediary. Moreover, it is not necessary that solutions requiring Blockchain are needed in all the sectors despite much hype taking place currently concerning this technology [59]. This is especially the case given the “lack of a systematic approach to understand Blockchain, its potential, and the development of convincing use cases [59]. This reality is based on the analysis of various scholars. For instance, Schuster argues that Blockchain-based systems are “unsuitable for transactions in traditional assets, unless design choices are made which render the use of the technology pointless” [143]. Nonetheless, Blockchain in Trade Finance is extremely useful since it (1) removes the need for laborious paperwork and verifications processes, since this is immediately available to all parties; (2) “radically improve operational efficiency both in the application process and execution phases for all stake; (3) alleviate risk associated with Trade Finance by creating a transparent data sharing infrastructure; and (4) make the offering of Trade Finance easier, cheaper and more widespread, driving competition in the Trade Finance sector and increasing business for smaller banks, while improving access to Trade Finance for businesses” [28].

The above has analysed the advantages of using Blockchain in Trade Finance, in the context of specific Trade Finance instruments such as letter of credits, highlighting the specific benefits for these instruments [20]. Despite the above-mentioned benefits of the use of Blockchain in Trade Finance, the actual application of Blockchain on a large scale in this field is yet to be seen [57].

## Risk and challenges of Blockchain Trade Finance

Scholars have analysed the challenges facing the effective use of Blockchain in Trade Finance. For instance, Rebecca M. Nelson stated that “Many cryptocurrencies are considered to be volatile, create a host of consumer protection and illicit finance concerns, face an uneven global regulatory environment, and require sizeable energy resources for the associated computations. Some sceptics allege that many cryptocurrencies are effectively a Ponzi scheme and primarily finance illicit activities” [144]. The following sections will provide an overview of the main issues that may require regulation.

A. Technical challenges

Currently, there are limitations facing the performance, scalability and efficiency of Blockchain technology given their complex designs and the existing limits of their transactional capacity [18]. The scalability challenge is one of the most important challenges as there are a lack of resources “to quickly and cheaply process information exchanges across an international network”, while public Blockchains prioritized decentralization and security instead of scalability [127]. Decentralization opens the door for money laundering, fraud and tax evasion while simultaneously making it difficult to supervise and manage operations [156, 157, 178].

There are concerns related to two conflicting values: security and privacy. On the one hand, officials want to “foster public policy goals of financial stability, investor protection, customer protection and market integrity, and to guard against illicit activities”. On the other hand, the private sector wants to ensure the privacy of the data for commercial and legal purposes, while individuals also have their legitimate reasons to maintain privacy [18]. The need to address questions of privacy and security is essential for other factors. These include inter alia the high dependence on third-party technology providers where common rules on security among the participants are extremely difficult to agree upon; the “potential risk of concentration or dependence on a single consortium or a single point of failure” [119]; the incentives for internal and external fraud such as organized crime [119] potential hacking of the system, the loss of data or the negative impact on the identity of an individual on the network [60]; and war, poverty and more [20, 159].

Blockchain is interoperable where there is a need for linking infrastructures, databases and technologies where questions of trust are being raised especially concerning the party that shall be responsible for the transfer of assets and information to the Blockchain or across chains [18]. This is because there is no uniformity in distributed ledgers as ledgers cannot communicate with each other due to the lack of interoperability [33]. Interoperability and compatibility need to be addressed given the need to integrate the Blockchain-based model with the current Trade Finance system [71].

There are high development costs and challenges resulting from the integration of several disciplines of the newest technologies [178]. When it comes to software updates, the absence of a centralized authority making the shots without the consent of users may lead to further complexities as some software updates require consensus, which is hard to reach. In some cases, the absence of consensus led to a split in the Blockchain [18]. Moreover, it takes time to add a new block to the Blockchain (around 10 min) and the block’s capacity is limited (1 MB), while 8 transactions per second are allowed online. This is in contrast, for instance, to Alipay that allows the conduct of thousands of operations per second [178].

There is a lack of understanding and acceptance of the Blockchain especially as it is not easy to identify effective and actual Blockchain financial products. Besides cryptocurrency, the rest of the Blockchains require further development and improvement [178]. This is why companies currently using Blockchain are still not overwhelmingly using it for critical functions [18], particularly as the novelty of the technology also impacts its development negatively [33].

Other challenges include poor user experience occurring because of the complicated relationship between the various stakeholders (e.g. banks, carriers, importers and exporters). There are also specific disadvantages affecting each stakeholder. The importers must pay an expensive charge of the financial instruments, and the issuance of such instruments is more difficult, while there is a lack of physical cargo inspections, since the payments are made upon the presentation of the documents. Banks have to deal with the possibility of disruptions in transactions and the “labor-intensive process of paper-based administration”, while the risks are mainly on the buyer’s bank. The logistics carriers need to deal with the limited transaction speed and the waste of computational resources as well as the security risks [20].

The main challenge is addressing all these challenges in a context of a constant increase in the number of trade entities, number of transactions and databases [71].

B. Legal challenges

The legal field is progressing slowly when it comes to emerging technologies such as DLT [100]. These challenges from a legal perspective are related to technology and standardization which must be addressed before the obligation becomes effectively used. In that sense, the new rules that ought to be adopted to address the technology should not restrict its use especially as the various barriers mentioned above remain to be tackled [57]. Currently, the regulatory frameworks concerning the use of DLT in Trade Finance are incomplete and require upgrade at the global and national level, as the laws need to be efficient in a manner allowing innovation [127]. There are also legal limitations as regulatory frameworks potentially may not adopt rules applicable to DLT [122]. Hacker et al. consider that the main legal challenges are to determine how Blockchain applications interact with neutral and general laws and to figure out new legal tools when existing laws are insufficient.

New technologies or platforms challenge existing regulations of Trade Finance [73]. In this context, there are also concerns that users may not get to participate in making the rules which may be made by a technocratic minority [117]. Authorities and policy-makers need to support the user, given the user’s lack of knowledge especially the technical knowledge by enacting legislations on vendors and service providers. Yet, regulations must be balanced to avoid the elimination of innovations [138]. There are proponents and opponents of regulating DLT, where some scholars advocate for its regulation due to various concerns, especially as regulating does not mean limit or reduce the development of the technology. Others worried that stricter regulation may hinder innovation in the field, stating that “legislating Blockchains makes no legal sense, especially since Blockchain are defined by their functionality” [117].

DLT does not need jurisdiction, yet laws must recognize DLT as a transaction, causing an ownership change. In that sense, legal frameworks must develop for the regulation of DLTs to provide assurances to all the stakeholders involved in the transaction where several countries have developed such frameworks [38, 173]. States can intervene to regulate the actors and the activities carried out in their territories [117]. Yet, there is potentially a problem of jurisdiction as various Blockchain nodes will be located in different locations. The laws in these locations may conflict with each other, while there may be a lack of enforcement of digitally signed contracts. Moreover, specifying the jurisdiction in which a potential dispute may be resolved is essential [119]. Yet, DLT must also comply with rules from day one, unlike in the early days of DLT with some instances of it being operated outside the legal framework [173].

Legislators are facing great problems when it comes to the regulation of DLT in Trade Finance as a result of the lack of awareness of the technology in the various sectors where it is expected to be implemented, in addition to a lack of understanding of how such technology works [57].

In terms of governance, there is a lack of a central authority that can govern and be held liable especially when issues arise, such as “who can participate and each participant’s role, what happens if someone loses their private key, and expelling non-compliant members” [119]. In contrast to a traditional centralized authority, the laws cannot be implemented easily and rapidly [29], as a consensus about the ledger is needed, which requires a higher coordination and further effective solutions to create legal and technological trust [175]. Other challenges include standards as “Blockchain-based Trade Finance service will require the implementation and adoption of a data standard and protocol that will populate the specifics of the process”. Moreover, there are further questions once a standard is created. For instance, “how will buyers, sellers, and any required trusted third party/intermediary, interface to the network?” [56].

All the above-mentioned challenges must be addressed by various nations, government agencies and industry stakeholders, which requires a certain level of collaboration that is extremely difficult to reach and takes time to develop [33]. Finally, it is difficult to establish global collaboration over the DLT technology due to cultural diversities and various political systems. A recognition of this technology in the legal field globally requires a consensus among all implementing nations [178].

## Qatar

DLT is not explicitly regulated by Qatari law, but types of its applications are rather covered within the scope of other legal regimes, mainly the cyberlaw of 2014 [24] and the Data Privacy Protection Law of 2016 [89]. Even before the emergence of DLT, obtaining Trade Finance in Qatar was, and still is, an extremely complicated process that is inconsistent and conducted through policies adopted by the Qatari financial institutions. The process was complicated by local issues such as addressing money-laundering threats and potential terror-financing activities [156]. From a legal perspective, Qatar lacks a “set framework for the provision of Trade Finance facilities”… as specific requirements are imposed on each business by individual providers where these requirements may slightly differ “for each type of Trade Finance facility on offer”. Individual Trade Finance providers determine the nature and extent of documentation and “is a function of each banks’ respective risk profile, internal discipline and commercial strategy and loan posture” [28].

The principal Trade Finance instruments used in the country are “letters of credit, letters of guarantee, bills of exchange, promissory notes, cheques, documentary bills (documents against payment (DP)/documents against acceptance (DA)) and export bills for collection (EBC)” [28]. The main rules regulating Trade Finance are in Part 4 Chapter 6 of Qatar’s Commercial Code [2]. Two other actors play an important role in the regulation of Trade Finance in Qatar: Qatar Financial Centre (QFC) and Qatar Central Bank (QCB). Dahdal, Truby & Botosh summarized the current situation of Trade Finance in Qatar.

The Trade Finance process in Qatar is presently shaped by the operational requirements of financial institutions with the broad outlines of the Commercial Code and slightly more detailed provisions of the QCB. The law provides clarification pertaining to definitions, rights and liabilities arising from Trade Finance instruments. The commercial application processes, across providers, remain tedious and costly. The organization of various documents and attestations as to their validity and currency present, and have historically presented, obstacles to the smooth flow of Trade Finance supported trade in Qatar and globally [28].

The main application of DLT that has been regulated rigorously in Qatar is of digital currencies. Many digital currencies depend upon DLT, and the rigid governance of digital currencies may have a dissuasive effect on DLT development generally. Nevertheless, there have not been signs that Qatar wishes to restrict DLT in Trade Finance or generally outside of its application by digital currencies.

In 2018, the QCB banned Bitcoin trading through Circular No.: 6/2018. It considered it as an illegal and high risk activity and urged “all banks operating in Qatar not to deal with Bitcoin, or exchange it with another currency, or open an account to deal with it, or send or receive any money transfers for the purpose of buying or selling this currency” [134]. It also imposed penalties on those violating the circular [134]. This was the first warning made to the financial institutions [4]. More recently in 2020, Qatar Financial Centre Regulatory Authority (QFCRA) declared that “crypto asset services may not be conducted in or from the Qatar financial center” while imposing penalties on the firms that provide such services. The ban includes “the exchange or transfer of virtual assets, or the exchange between virtual assets and fiat currencies” [67, 133]. This very much reduced the options to trade with digital currencies in Qatar.

Qatar is currently focusing on the development of a FinTech strategy to incorporate digital innovations within the financial sector including the development of Blockchain-based technologies [154]. The Qatari government adopted a cautious approach addressing the risks by banning specific activities, while acknowledging the potential benefits specifically of Blockchain as form of DLT, by including it in its FinTech strategy. One can understand the balance that the Qatari government is trying to strike by recognizing simultaneously the existing risks and opportunities. Several scholars have stressed the potential benefits of DLT for the Qatari system such as the financial and multi-sectoral utility benefits as highlighted by Truby with “skilled job creation, investment and wealth creation” and “considerable advances in security and applications that can produce innumerable functions to benefit society, industry, and governance” [158]. Moreover, in addition to seeing DLT in Trade Finance as a means to drive “the economic diversification policies established by in Qatar Vision 2030”, Dahdal, Truby & Botosh also argue that the Qatari “legal framework is capable of supporting the introduction of Blockchain as a technical data retention and sharing platform with little or no need for specific Blockchain-focused regulations” [28].

This technology could be used in Qatar in the supply chain-intensive industries, and the management of big infrastructures in sectors such as hospitality or agriculture, in addition to managing organizations such as schools and hospitals, while helping in the maintenance of roads and bridges. In the liquefied natural gas industry, Blockchain “would help prevent illegal manipulation or systems tampering, and other forms of cybercrimes”. DLT can even support the Qatari residence permit system [5] and facilitate the potential development of new renewable energy markets [149].

## Applying a cautious approach to Trade Finance via Blockchain technology

Given that Qatar’s strategy is seeking to benefit from the use of Blockchain technology while avoiding the risks, a flexible approach is required to strike the balance sought. The following sections will provide several suggestions that can be combined into a compressive plan.

- Embracing international regulations and standards

The transboundary nature and risk of DLT require the adoption of international regulations, harmonization of existing rules and coordination between countries. This is extremely important given the existing shortcomings of regulating this field at the domestic level, as highlighted previously in the legal challenges section. Still, there is a lack of consensus at the international level in this regard [102]. This is despite the need for strong synergies between different stakeholders of the technology in the general framework of an effective cooperation mechanism, allowing each stakeholder to perform its tasks in a coordinated manner [178]. International cooperation among the various governments is needed to agree on international regulations [121], despite the challenges facing international cooperation due to divergent countries’ laws, approaches and interests [90]. Standards and regulations must be established to ensure the appropriate utilization of DLT technology and the realization of its objectives. Regulations must be constantly improved [178], while legal frameworks adopted before the emergence of DLT may need to be updated or new rules adopted [18]. Global coordination is essential to protect the system and ensure that its benefits are realized where such regulations and standards need to be flexible to allow innovation [60], especially as DLT is expected to disrupt the legal system [109].

In this context, the race for the adoption of international regulations and standards has begun. For instance, Salmon and Myers argue that a global agreement on digital economy is needed to address issues like the taxation of Blockchain and other forms of DLT, as various jurisdictions would have different rules. This would result in an uncertain tax environment which could deter investors and innovation [141]. In contrast, Fyrigou-Koulouri calls for the development of “a minimum standards/principles voluntary framework” that is non-binding, which would encourage states to accept it [60]. She argues for the development of an international non-binding framework that includes various elements such as a “… principle with respect to definitions, legal power, legal rights, and results of every feature/action in the Blockchain; architecture standards for Blockchain as guidance to both developers and users; privacy and security standards… [60]”.

There are other issues that international rules should also address such as human rights protection, and the type of international organization to regulate this field. For instance, it has been suggested that the UN as a result of its experience and organizational independence can regulate Blockchain—particularly the UN Commission on International Trade Law (UNCITRAL) [124]. In fact, there are studies examining the potential implications of Blockchain technology for the UNCITRAL Works [147]. At the same time, an International Association for Trusted Blockchain Applications (INATBA) was created in 2019 to act “as a global forum which brings together developers and users of distributed ledger technology (DLT) with regulators and policy-makers from all over the world” [25].

Besides the adoption of new laws, an analysis of the interplay between existing global regulatory frameworks and DLT is taking place. Guillaume argues that international private law rules must apply to Blockchain operations connecting this framework to national legal orders, to make sure that a legal framework applies to Blockchain transactions [70]. Meanwhile, the interplay between dispute settlement resolutions and potential Blockchain-based cross-border commercial disputes [51], and the impact of existing international regulatory frameworks such as international economic law on Blockchain is being examined [123]. Such assessment led scholars like Razon to suggest the application of the General Agreement on Trade in Services (“GATS”) to Blockchain as it “provides a solid foundational framework for services supplied by participants on Blockchain, even if, unsurprisingly, several grey areas exist” [136].

In terms of standards, there are calls for the establishment of global standards for DLT to encourage international participation. Since 2016, the development of international standards on Blockchain was initiated by the International Organization for Standardization (ISO). Standards Australia was selected to lead this task [114] after submitting a request to the ISO [72]. International organizations and institutions like the International Organization of Securities Commissions (IOSCO), the Financial Stability Board (FSB) and countries securities regulators have been making statements to determine the best way to regulate DLT. The emphasis of all the statements is on international coordination despite the existence of various regulatory and political systems resulting in different laws related to investor and consumer protections globally [18]. Organizations such as the Financial Action Task Force (FATF) issued Guidance for a Risk-based Approach to Virtual Assets and Virtual Asset Service Providers; the FSB issued high-level recommendations for stablecoins, while the IOSCO issued a report on the regulation of cryptocurrency exchange platforms [102]. Standards already in place making an impact in “financial Blockchain’s consensus model, private data encryption, intelligent contract operation, and scalable data model” [148] include the Chain Open Standard, as a result of cooperation between a Blockchain technology supplier called Chain and the most important financial institutions like Citigroup, Fidelity, First Data, etc. [148].

Examples of other institutions working on the development of standards include the Organisation for Economic Co-Operation and Development (OECD) that “committed to help governments to find experts and practitioners to engage with, and to identify and share best practice for governments managing and using Blockchain”. The OECD is conducting research on this field to address the various questions and challenges. It established the Blockchain Policy Centre to that end. The International Chamber of Commerce, the Trade Financial Global and the World Trade Organization are also conducting research on Blockchain. The Global Blockchain Business Council launched during the 2017 World Economic Forum is playing a role in addition to other governmental and nongovernmental organizations [25].

Not only are international regulations and standards important for DLTs, but it seems that this technology is also seen as a means for enhancing international governance. De Filippi argues that “Blockchain technology could help build a more resilient and trustworthy international governance system through a distributed coordination infrastructure, allowing for a multiplicity of stakeholders (governments, corporations, NGO and civil society organizations) to collaborate in order to solve some of the most pressing global challenges of an increasingly interdependent world” [30].

International regulations and standards that can be used are not only the ones related directly to Blockchain technology. International organizations have developed strategies to implement reforms and enhance technology-based solutions. These generally require (1) diagnostic, (2) gap analysis and market surveys, (3) international standards + comparative analysis, (4) recommendations, (5) pilots, (6) assessments, (7) revision and correction. For instance, the UN is adopting various decisions and documents related to the use of technology for sustainable development, trade and economic development [166, 168]. In fact, the UN adopted a Strategy on New Technologies in 2018 [164]. Reports and resolutions on technology and innovation such as Resolution 2019/25; science, technology and innovation for development have also been adopted [169]. Similarly, other organizations like the IFC, EBRD, ADB and so on adopted reports, recommendations and documents on technology-based solutions [9, 39, 81, 82]. For instance, the World Bank has adopted numerous policy papers addressing DLTs, Blockchain and the problems that emerge as a result providing recommendations on how to secure DLT transactions from a legal perspective. These papers highlighted three main concerns: (1) “the tension between the social benefits related to financial innovation and its possible uses for purposes of regulatory arbitrage; (2) the rapid diffusion of DLT might entail unintended consequences that threaten the smooth functioning of the financial system: (3) the lack of regulation stifles, rather than facilitates, the diffusion of forms of financial innovation that benefit society” [86, 151, 152, 153].

International rules and standards are expected to play an important role in the Blockchain field. Therefore, states like Qatar must benefit from the developments occurring and incorporate them within the Qatari legal system.

b. Replicating foreign national rules that are appropriate and innovative

Regulators at the national level are responsible for directing technological innovation towards the specific objectives connecting socio-institutional systems to techno-economic reality [55]. In the context of DLT, regulators are responsible for the adoption or the expansion of new laws [132]. The laws related to DLT are those concerning the development of the technology, the control of the behaviour of the various stakeholders and its use [148, 176]. The importance of the regulator’s mission lies within the fact that those deciding the rules of use of DLT will have the power and control over the outcome. Hence, it is important to have national authorities regulating this field and not leaving a vacuum to start-up enterprises, users and global corporations [177]. In this context, Morton argues that policy-makers and regulators must have a new mindset “to create uniformed regulatory bodies, policies, and enforcement protocols to be able to police illicit activities that arise from cryptocurrency usage” [121].

There are existing regulatory principles at the national level that can apply to DLT as nations are already looking within their laws for potential rules and principles that can contribute to the regulation of this technology [141]. The national rules that can be adopted are not only related to DLTs. There are already national legal frameworks that address, for instance, money laundering, tax evasion, intellectual property rights and so on. The development of a new technology to conduct these actions does not change the legal framework governing these fields but rather specific regulations addressing the technology itself are required [60]. Yet, there is confusion when it comes to the regulation of DLT as it is not clear what laws to apply, the kind of government intervention needed and tax to impose and legal status of the various types of DLTs. Nations, especially developing nations, will likely be looking at the way the US, EU and Canada regulate this field, while other countries such as China and Japan are also very important in this regard [121].

The EU is very active in the DLT field and particularly Blockchain, leading to the establishment, for instance, of Blockchain Observatory and Forum in 2018 “a stakeholders engagement platform which monitors key initiatives in Europe allowing to connect European and global expertise, and hence the gathering and sharing of knowledge on the subject” [25]. A European Blockchain Partnership (EBP) was also created in 2018 through a formal declaration “defining a policy agenda for Blockchain by identifying critical regulatory areas such as smart contracts” and “building a European Blockchain Services Infrastructure (EBSI) which aims to deliver EU-wide cross-border public services using Blockchain technology” [25]. The European Commission as well as other EU institutions is focusing on the regulatory and legal aspects of Blockchain-related technologies [25]. For instance, the European Parliament adopted non-legislative resolutions on Blockchain such as the 2018 Resolution on “Blockchain: A Forward-Looking Trade Policy” [25]. Yet so far, the EU did not adopt regulations expressly regulating Blockchain but rather established initiatives in this regard. Rather the EU adopted proposals and initiatives concerning DLT as well as cover cryptoassets or digital assets. For instance, the EU adopted a Proposal on a pilot regime for market infrastructures based on distributed ledger technology on September 2020 [44]. Further developments and work on Blockchain technologies are being conducted by the various EU institutions [25], resulting, for instance, in the release of the Proposal for a Regulation on Markets in Crypto-assets (“MiCA”), by the European Commission in September 2020 [19], in the general context of the European initiative for the development of EU Regulatory Framework for cryptoassets [41]. Such proposals in addition to new ones that will be adopted as a result of EU’s effort in addressing Blockchain technology in various fields [46–49] can serve as guidance even though they are not directly related to Blockchain in Trade Finance.

Several European countries adopted national rules or approaches for the regulation of DLT, including Blockchain or the regulation of cryptoassets or digital assets. These include France, deciding that “only active participants—those actively inputting data into the system, and not mere ‘nodes’ or ‘miners’ providing verification of transactions to the platform—are responsible as data controllers” [141]. In fact, some countries like France and Poland adopted specific regulations addressing the use of Blockchain especially in the financial sector. The most progressive legislation is Liechtenstein in adopting the Liechtenstein Act in 2019 [25, 63, 104]. Small countries like Malta are adopting rules related to DLT. In 2018, it enacted three new laws: the Malta Digital Innovation Authority Act [111], the Innovative Technology Arrangements and Services [88] and The Virtual Financial Assets Act for regulating the cryptocurrency and Blockchain ecosystem [125, 174]. Moreover, some developing countries have also adopted rules. For instance, Thailand adopted two decrees in 2018: “Operations of Digital Asset Business (Royal Decree on the Digital Asset Businesses B.E. 2561)” and “Tax Implications on Income Earned from Digital Assets (Royal Decree of the Amendment to the Revenue Code)” [125]. Existing general rules applicable to DLT including Blockchain, specific regulations related to Blockchain as well as to cryptoassets or digital assets can serve as guidance for the adoption of new rules on Blockchain in Trade Finance.

It is worth mentioning that states adopted various approaches and strategies when it comes to the regulation of DLTs including Blockchain as well as cryptoassets or digital assets. In this context, there has been a specific focus on regulations addressing DLT applications in several places. For instance, the US federal regulators and law enforcement adopted different classifications of Bitcoin under existing federal laws where even state and federal courts differed in their rulings. In the ruling in *Florida vs Espinoza,* Bitcoin was considered a property in contrast to the 2014 US federal ruling in the USA v Faiella, aka “BTCKING” and Charlie Shrem, where Bitcoin is considered as money or funds [128]. Other nations granted different legal status to Bitcoin. The German Federal Financial Supervisory Agency considers Bitcoin to be a financial instrument called a unit of account in contrast to the Central Bank of Slovenia that does not consider it to be a financial instrument. The Bank of Finland claims that it is more close to a commodity, while the Dutch Central Bank argues that Bitcoin fails “to fully fulfil the three functions of money: medium of exchange, store of value and unit of account” [128]. Hence, there are various approaches globally when it comes to considering the legal status of Bitcoin, where different financial regulators have either remained silent, argued that it does not have a regulatory scope, restricted its use, warned against its use and even banned it [128].

The existence of numerous initiatives and attempts by various nations to regulate DLT applications as well as cryptoassets or digital assets at the national level should serve as a model to the rest of the states seeking to reach similar objectives. Qatar can benefit from these rules especially the sophisticated ones like EU regulations and incorporate the rules deemed appropriate within its domestic framework for the regulation of Blockchain in Trade Finance even though they are not directly regulating this field. Yet, one has to keep in mind that the objective is not to simply replicating foreign national rules or “legal transplants” but rather using these rules and amending them to consider the Qatari context to avoid the transplant of vague and inappropriate notions and provisions especially as in many instances such replication is impossible [66, 79, 106].

c. Applying sandbox regulations to Blockchain products and services

The concept of the regulatory sandbox emerged from the UK with the goal of providing more space for innovation [72]. The UK developed the regulatory sandbox regime “providing an incubator for financial technology firms to test their products under supervision and gain access to finance without the costs and restrictions of the usual regulatory requirements” [160]. Such controlled platforms are suitable especially for financial firms that are seeking to test risky products. This is how the UK became a global leader in FinTech start-ups. This success led other states to develop their own sandbox regimes that are similar to the British one [160]. Currently, “multiple jurisdictions provide a form of ‘beta testing’ for financial technology start-ups, where firms may test their financial services technology and other financial products under supervision of the financial services authorities” [160].

The use of regulatory sandboxes allows the testing of services with customers in a safe and supervised environment, where such testing will not result in regulatory consequences. Through this procedure, regulators have the possibility of understanding the new technology and cooperating with the industry stakeholders with the aim of developing regulations that encourage innovation in this field, protect the users and allow the development of technology solutions simultaneously [141]. It is worth mentioning that nations developing sandbox regulations are collaborating to allow financial firms to access each other’s market where, for instance, the UK and Australia established the “FinTech Bridge” agreement. What is more, a global sandbox is being developed with the contribution of various financial regulators in the context [160] of the Global Financial Innovation Network (GFIN) launched in 2019 [65]. Regulatory sandboxes can take the form of a FinTech Supervisory Sandbox, for instance [21]. A sandbox express was created in Singapore allowing the conduct of activities regulated by the monetary authority [37].

The use of sandboxing increased especially after the financial crisis to strike a balance between regulators’ desire to attract and enable innovation and the need to preserve and apply existing regulations in the financial services industry. The main benefits of sandbox regulations are: (1) helping the “regulator to revise and shape the regulatory and supervisory framework with agility”; (2) helping to “coordinate and align the fast growing pace of FinTech firms with compliance and regulation, at the same time without compromising on customer security”; and (3) “attracting investors such as banks, private equity and venture capitalist for investment in FinTech firms” [6]. Other important functions are: “(1) reducing the time and cost of introducing innovative products or services to consumers; (2) analysing the risks of new business models and underlying technologies; and (3) assessing if the regulatory approach is balanced for mitigating those risks while enabling innovation in their markets” [37].

The existing definitions and traditional functions of regulatory sandboxes have been criticized. For instance, Kera argues that “sandboxes should offer rich feedback on the type of issues, hopes, and fears the various stakeholders experience while engaging with the new service, rather than only a quick recipe on how to enable adoption without too many regulations” [98]. Sandboxes may create problems such as leading to a lack of transparency and favouritism as “it's often hard to tell exactly what waivers or exemptions are being granted by the regulator to private firms, or whether the regulator is providing other means of support”. Also, often it is not clear why a firm was included in the sandbox in contrast to others [115]. It is important to mention that a regulatory sandbox cannot eliminate all the existing risks which is why usually safeguards are implemented to address any potential failure and protecting the financial system [35].

Regulatory sandboxes are used in fields where modern digital technologies may result in new products and services including Blockchain [137]. Already the UK, Australia, the USA, Hong Kong, Malaysia, Singapore, Switzerland, Thailand and United Arab Emirates have either implemented or are examining the idea of using regulatory sandboxes for Blockchain [125]. Examples of regulatory sandboxes applicable to Blockchain include the Hong Kong “two-staged sandbox for cryptoasset platform operators” which aims to regulate cryptoasset exchanges and is considered as an innovative mechanism [77]. The UK Financial Conduct Authority FCA’s regulatory sandbox has accepted companies using DLT or deal with cryptoasset services which allowed the FCA to work with these companies to understand the business model and their potential impact on the market. Abu Dhabi Global Market has been using sandbox regulations to work with cryptoasset firms to understand cryptoasset products. Other countries are even more progressive as they established sandboxes focusing only on cryptoassets and DLT. Examples include the Bank of Lithuania’s LBChain allowing the bank of Lithuania to understand the technology and “creating collaboration and experience-sharing opportunities for the firms accepted in the sandbox” [14].

Guo & Liang suggest the use of regulatory sandbox regulations for Blockchain [72]. Regulatory sandboxes may offer opportunities for improvements of the use of modern technologies in the financial sector including Blockchain [162]. There are numerous challenges facing the deployment and use of Blockchain technologies which were addressed in previous sections. The existing legal and regulatory environment does not facilitate the dissemination and further development of Blockchain, especially as different laws apply depending on the jurisdiction [146]. These challenges render the idea of governance by Blockchain extremely difficult especially for regulators, given the existing risks. Sandbox regulations offer a rather pragmatic model addressing these challenges by having actual experiments on the technology in safe and controlled environments [98]. In this context, Kera argues that “regulatory sandboxes for emerging technologies, such as Blockchain, should function as such trading zones between code and regulation rather than safe spaces for innovation without regulation” [98].

Regulatory sandboxes for DLT technologies including Blockchain technologies and cryptoassets or digital assets are emerging. Regulators need to embrace this new approach based on test-and-learn philosophy [129], including Qatar, that is seeking to pave the road in DLT-based FinTech. This does not mean to embrace all the above-mentioned examples of regulatory sandboxes but rather examine whether they are appropriate, whether certain elements and aspects can be used and add these elements and aspects to a new regulatory sandbox to be developed for testing Blockchain technologies in Trade Finance.

## Qatar’s multi-layered governance approach to the regulation of DLT

The above analysis highlighted different available methods to regulate emerging technologies such as Blockchain as a form of DLT. Given the recent nature of the technology, existing technical and legal challenges as well as the need to strike a balance between the regulation of potential Blockchain risks and reaping the benefits while fostering innovation in this field, the authors are of the opinion that Qatar can adopt a multi-layered governance approach to the regulation of DLT. Qatar should benefit from existing international rules and standards as well as national rules while remaining flexible and but also supportive of DLTs by applying sandbox regulations.

### Embracing international regulations and standards

Qatar’s recourse to international law can be seen especially through the 2017–2021 blockade imposed by the Kingdom of Saudi Arabia, the United Arab Emirates (UAE), the Kingdom of Bahrain and the Arab Republic of Egypt where Qatar questioned the legality of the measures taken. This follows the approach of Qatar to engage with international law and comply with international rules and the various courts decisions [7, 97]. This can be seen, for instance, through Qatar’s accession to various international treaties and instruments, respect to the rule of law as enshrined by the UN, the belief in the importance of international law as a “source of stability, security and safety for all peoples of the world” and the importance given to international cooperation [163]. This does not mean that the country agrees with the content of all existing conventions as some reservations were made similarly to other states [4, 17]. The main reasons for this approach are maintaining the security and stability of the country, facing geopolitical threats and challenges, expending the influence of the country as a regional player and becoming an important actor at the international scene [99]. The blockade only accelerated Qatar’s recourse to international law especially as Qatar was able to secure judicial success [64]. Given this reality, Qatar can benefit from this approach and its soft power to influence the development of international rules and standards related to DLT as well as profiting from existing and future laws and guidelines to incorporate them within the Qatari legal system. With this, Qatar would comply with international law and standards and benefit from them simultaneously for the regulation of Blockchain domestically. For instance, similarly to its role in the adoption or taking part in existing international treaties, Qatar can participate in the negotiation process and adoption of a future international treaty on DLT, as previously suggested.

### Adopting existing regional and national rules that are appropriate and innovative

The European civil law model is the basis of Qatar’s legal system as this model was used in the Middle East because Egyptian Jurisprudence had a great influence in the early days on the development of laws and regulations of independent Arab states [28]. This meant that Qatar’s legal system is indirectly influenced by the French civil law that influenced Egyptian civil law [69]. In addition to French civil code and Egyptian jurisprudence, Qatar’s civil law is also based on Islamic law [23]. Moreover, British legal institutions were introduced with the British involvement in the country from 1916 to 1971, where the combination of political influence of Great Britain and the discovery of oil in 1940 facilitated the introduction of Western laws [74]. The current Qatari civil code is considered one of the most recent civil codes in the Arab world [75]. In fact, the constitution of the country was adopted in 2004 [58] as a provisional constitution was in place before [78]. Moreover, the European civil law model was the basis for the Trade Finance laws of Qatar incorporated in the Commercial Code mainly Part 4 Chapter 6 [28]. In this context, Qatar has been analysing best practices and laws of other countries for making further institutional development and adopting modern laws [78], especially as numerous laws of the country are based on other nation’s domestic rules. Similar situations should occur in the DLT context especially as numerous countries and regional actors are already adopting certain rules specific to Blockchain. In particular, Qatar should benefit from the current initiatives and rules of the EU, various European countries, the USA and Canada related to DLTs, cryptoassets or digital assets or specifically addressing Blockchain in Trade Finance while monitoring developments taking place in Asian countries such as China, Singapore and Japan. This does not mean that the state should embrace all the developments but rather select the most appropriate and innovative ones that would fit the Qatari legal system without replicating the terms but adopting new rules suitable to the Qatari context [116].

### Applying sandbox regulations to evaluate the application of Blockchain

There are risks and threats associated with the emergence of new technologies that present a great challenge from a regulatory perspective. The main challenge is the regulation of the risks while fostering innovation in that same technology [112], especially as unknown and unforeseen risks usually constitute a part of the innovation process. Regulators have two options: either rely on existing rules or adopting new regulations. Yet, using flexible tools to emerging technological fields is more appropriate [140], as these technologies “tend to have many diverse applications and forms, are used in many different industries and contexts, and present a multitude of different and often hard-to-quantify risk and benefit scenario” [113].

Qatar’s willingness to invest and use flexible tools to regulate DLT-based emerging technologies such as in FinTech has been noticed in recent years with the launch of regulatory sandbox by the QCB [52], along other initiatives such as the launch of the Qatar FinTech Hub (QFTH) [95]. The objective is to foster computer security in terms of trust and reliability [1], as part of Qatar’s FinTech strategy [135]. Corporations were invited by the QCB “to identify their operations to begin testing their products and (including FinTech corporations targeting virtual payments)” [130], since the QCB is seeking to develop a sandbox environment [150]. In fact, there also a discussion about the use of sandbox regulation for Islamic FinTech in the Qatari context [105]. Given that Qatar is already using the regulatory sandbox as part of its FinTech strategy, it would be appropriate to adopt the same legal tool for the regulation of DLT technologies. This would allow companies to experiment in this field in the Qatari context in a controlled environment responding to the concerns related to the risks and challenges of DLT while providing a room for innovation. Such sandbox should be based either on existing financial sandboxes like the global sandbox that is being developed or the ones that are being developed by various countries such as Singapore, the UK and Lithuania. Moreover, elements from existing regulatory sandboxes that are not related to Trade Finance or Blockchain in trade should also be used if they are appropriate.

The three suggestions described above should be used simultaneously by the regulatory authorities of Qatar to provide the best regulatory environment for the application of DLTs in the country. This will allow maximum flexibility to foster innovation while controlling the risks with appropriate and sophisticated regulations.

## Recommending specific rules and standards to apply to DLT in Trade Finance in Qatar

This section will provide concrete examples related to the regulation of DLTs at the international, regional and national level as well as specific sandbox regulations being developed to that end.

### Based on international regulations, standards and non-binding instruments

There are various international legal fields and sectors that are being examined to provide solutions for the regulation of DLTs. While some of them can provide benefits for the Qatari system, others are still underdeveloped where Qatar can push for their development.

The main one is international private law given that DLT constitutes a cross-border transaction between private individuals [179]. Yet, so far, states did not attempt to “unify the rules of private international law applicable to digital activities via a multilateral international convention” as it is extremely difficulty to establish the location of the DLT transaction [70]. Qatar according to its approach to international law can push towards international private rules applicable to DLTs especially in Trade Finance. At the same time, there are suggestions for the use of Blockchain in the general framework of the conventions of the Hague Conference on private international law thus using Blockchain to facilitate the implementation of international conventions [139]. Similar discussion is taking place with regard to the positive and negative impact of DLT on international trade law [61], Blockchain arbitration [12], maritime law, etc.[62]. Qatar could also participate in this process to understand the pros and pros of DLTs to facilitate the implementation of international treaties and play a leading global role.

Similarly, Qatar can benefit from current suggestions related to the regulation of DLTs globally. These include the potential replication of the UNCITRAL Model Law on Electronic Commerce of 1996 especially the principles of non-discrimination, technological neutrality and functional equivalence [70, 161], as well as the possible replication of provisions of the UNCITRAL Model Law on Secured Transactions mainly Article 12 [167]. Articles adopted in other instruments that are being suggested to apply include Article 12 of the United Nations Convention on the Use of Electronic Communications in International Contracts. This article is seen as potentially useful for the interpretation of smart contracts in Blockchain [70, 172]. Given the impact of Blockchain in various field, a debate is taking place concerning the implementation of rules from existing conventions that are interacting with Blockchain. These include conventions related to copyright law since “some features of Blockchain technologies—scarcity, trust, transparency, decentralized public records and smart contracts—seem to make this technology compatible with the fundamentals of copyright”. Examples include the Berne Convention for the Protection of Literary and Artistic Works; the 1994 Agreement on Trade-Related Aspects of Intellectual Property Rights; and the 1996 WIPO Copyright Treaty [15]. Qatar can assess the various suggestions made such as the ones described here to regulate DLTs.

Qatar can also benefit from the efforts made by various international organizations for the development and enhancement of technology-based solutions including solutions to Blockchain technology. Examples include the work of the UN addressing Blockchain in the context of trade facilitation; sustainable growth; and its general application to the UN system where various recommendations are being made in this regard [36, 165, 170, 171]. Meanwhile, EBRD has developed its own version and understanding of smart contracts to be used in the Blockchain sector providing recommendations for policy-makers with regard the development of legal and regulatory frameworks related to smart contracts and its application to Blockchain including Blockchain in Trade Finance [40, 110]. Also, the IFC is addressing Blockchain and DLT in Trade Finance making recommendations and providing solutions especially in the context of emerging markets [83–85]. Qatar can benefit from the developments taking place be it via the various (1) diagnostic, (2) gap analysis and market surveys, (3) international standards + comparative analysis, (4) recommendations, (5) pilots, (6) assessments, (7) revision and correction taking place within international organizations.

Finally, ISO is leading in the development of Blockchain standards where more than 50 countries are participating in the development of these standards which is very beneficial as the people that need them are involved in their development [126]. The standards are: “(1) terminology and concepts; (2) overview of privacy and personally identifiable information protection; (3) security risks and vulnerabilities; (4) overview of identity; (5) reference architecture; (6) taxonomy and ontology; (7) legally binding smart contracts; (8) overview of and interactions between smart contracts in Blockchain and DLT systems” [120]. And indeed ISO is currently developing various international standards applicable to Blockchain [91] such as ISO/AWI 22739 Blockchain and distributed ledger technologies—Vocabulary [92]; ISO/DTR 23249 Blockchain and distributed ledger technologies—Overview of existing DLT systems for identity management [93] and ISO/DTR 3242 Blockchain and distributed ledger technologies—Use cases [94]. There are other actors adopting standards such as the Institute of Electrical and Electronics Engineers (IEEE) [80], while the various Blockchain standards are being analysed and compared [101]. Qatar can benefit from these standards and replicate those that are appropriate in the Qatari context.

### Based on appropriate and innovative regional and national rules

There are various regional and national actors addressing Blockchain technology. The focus here is on the main actors.

At the regional level, the EU has been addressing the topic of DLT regulation in recent years, although not directly but rather through various initiatives and legislation addressing DLT, as well as cryptoassets or digital assets. Most recently, in September 2020, the EU commission issued a legislative proposal on a pilot regime for market infrastructures based on distributed ledger technology laying down “requirements on multilateral trading facilities and securities settlement systems using distributed ledger technology ‘DLT market infrastructures’, which are granted with a specific permissions to operate” [44]. It established requirements for “(a) granting and withdrawing such specific permissions; (b) granting, modifying and withdrawing related exemptions; (c) mandating, modifying and withdrawing attached conditions, compensatory or corrective measures; (d) operating such DLT market infrastructures; (e) supervising such DLT market infrastructures; and (f) cooperation between operators of DLT market infrastructures, competent authorities and ESMA” [44]. The European Commission also issued a legislative proposal on markets in cryptoassets (MICA) for the regulation of cryptoassets [43] in the general framework of the commission’s Digital Finance Package [54]. The latter includes proposals for the regulation of various digital matters comprising a proposal for a regulation on a pilot regime for market infrastructures based on distributed ledger technology [44]; a proposal for the regulation of digital operational resilience for the financial sector that covers “crypto-asset service providers, issuers of crypto-assets, issuers of asset referenced tokens and issuers of significant asset-referenced tokens” [45]; and a proposal to clarify or amend certain related EU financial services rules [42]. These proposals follow previous attempts made by the commission for the regulation of Blockchain technology such as the Commission’s proposal for the regulation of Markets in Crypto-assets in 2019 [43].

At the national level, there are various approached adopted. For instance, Malta is one of the main countries that adopted regulations addressing DLT. The state established a Malta Digital Innovation Authority through a 2018 Act to “regulate and develop framework for innovation, such as framework concerning Distributed Ledger Technology (DLT), Blockchain technologies, smart contracts and the components of its development” [111]. Malta also adopted Innovative Technology Arrangements and Services Act the same year to “provide for the regulation of designated innovative technology arrangements referred to in this Act, as well as of designated innovative technology services referred to in this Act, and for the exercise by or on behalf of the Malta Digital Innovation Authority of regulatory functions” [88]. Finally, the state adopted the Virtual Financial Assets Act to “regulate the field of Initial Virtual Financial Asset Offerings and Virtual Financial Assets and to make provision for matters ancillary or incidental thereto or connected therewith” [174]. These regulations that regulate DLT can be used when appropriate for the regulation of Blockchain in Trade Finance in Qatar.

The US state of New York is currently proposing a Conditional BitLicense Framework “easing the process for businesses to enter the New York virtual currency marketplace and providing further clarity in a complex area of regulation” [76]. More recently and at the federal level, the Department of the Treasury’s Financial Crimes Enforcement Network (FinCEN) proposed a new regulation entitled Requirements for Certain Transactions Involving Convertible Virtual Currency or Digital Assets requiring “money service businesses (which includes, for example, cryptocurrency exchanges) to collect identity data about people who transact with their customers using self-hosted cryptocurrency wallets or foreign exchanges” [10, 32]. Still, the USA is far behind other nations; there is hope that the Biden Administration could pave the road in terms of Blockchain regulation [13]. Hence, even though the above-mentioned regulations are not directly related to Blockchain, specific provisions may be used by Qatar in case they are deemed appropriate for the regulation of Blockchain in Trade Finance.

Singapore is also seeking to regulate DLTs, in this case cryptoassets or digital assets. The country adopted the Payment Services Act [118] to “regulate traditional as well as cryptocurrency payments and exchanges” [87]. The Monetary Authority of Singapore (MAS) made the Securities and Futures Act “applicable for public offerings or issues of digital tokens” and issued a new Guide to Digital Token Offerings in 2020 [87]. More recently in 2020, MAS proposed new regulations for cryptocurrency consisting of four provisions: (1) “A harmonized and expanded power to issue prohibition orders; (2) An addition to existing AML/CFT (Anti-Money Laundering/Combating the Financing of Terrorism) regulations on cryptocurrencies; (3) A harmonized power to impose requirements on technology risk management; (4) Providing mediators, adjudicators and employees of an operator of an approved dispute scheme with statutory protection from liability” [87]. Similarly to the USA, these regulations although not regulating Blockchain directly can be used in case they were deemed appropriate for the regulation of Blockchain in Trade Finance in Qatar.

Given the importance and innovative approach that characterizes new EU as well as developed countries’ regulations to emerging technologies including DLTs as highlighted by the USA and Singapore examples, Qatari policy-makers could examine the various regulations and proposals mentioned above as well other regulations and proposals adopted by other nations such as the UK and Canada and replicate those that are deemed innovative and suitable in the Qatari context taking into account the local needs and markets.

### Based on existing sandbox regulations to evaluate the application of DLTs

Several sandbox regulations are currently in place or being proposed for the regulation of Blockchain technologies.

At the regional level, the Council of the European Union adopted in November 2020 the Conclusions on Regulatory sandboxes and experimentation clauses as tools for an innovation-friendly, future-proof and resilient regulatory framework that masters disruptive challenges in the digital age. The Council called for the use of regulatory sandboxes for the regulation of disruptive technologies highlighting their benefits mainly “advancing regulation through proactive regulatory learning, enabling regulators to gain better regulatory knowledge and to find the best means to regulate innovations based on real-world evidence” [26]. In this context, a European Blockchain Partnership (EBP) was created [34]. The EBP in cooperation with the EU Commission is seeking to establish a Pan-European Blockchain regulatory sandbox for testing Blockchain regulations concerning “data portability, B2B data spaces, smart contracts, and digital identity (Self-Sovereign Identity) in the health, environment, mobility, energy and other key sectors”. This sandbox will be operational between 2021 and 2022 [50]. As mentioned earlier, the European Commission proposed a pilot regime for market infrastructures based on distributed ledger technology in September 2020 to “further enable and support the potential of digital finance in terms of innovation and competition while mitigating the risks” [44].

At the national level, Japan is one example where a regulatory sandbox is utilized for the regulation of Blockchain. Accordingly, FinTech, IT or finance companies, both local and foreign, can apply to take part in the regulatory sandbox in Japan, in particular for projects related to artificial intelligence, big data and Blockchain. The testing period is 12 months [68]. The UKFCA has also allowed the testing of Blockchain technologies in the general framework of the regulatory sandbox established to that end [142]. The sandbox procedure consists of four stages: (1) submitting an application containing a business plan and describing “how it meets the sandbox’s eligibility criteria” [31]; (2) obtaining authorization by completing all the paper work; (3) testing over an agreed period [31]; and (4) exiting by having “agreed customer safeguards and exit plans to implement upon the test completion” and submitting “a report to the FCA detailing the outcome of the performed test(s) and next steps” [31].

While some of these regulatory sandboxes may be general in nature or applicable to the broad financial sector, Qatar could benefit from the experiences and developments taking place in other places such as the EU, UK and Japan to develop its own procedure and rules concerning regulatory sandboxes, and its application to digital technologies, such as Blockchain in Trade Finance, and adapting them to relevant Qatari context.

## Conclusion

DLTs are here to stay especially as several international financial actors see these technologies as a way to address the economic recession occurring because of COVID-19 [102]. Given the existing risks and potential shortcomings surrounding the use of DLTs including Blockchain in Trade Finance, suggestions were made to improve Blockchain transactions. These include, for instance, (1) increasing the number of participants; (2) adding to “Blockchain the ability to conduct transactions in electronic fiat money or in a digital currency, which is pegged to fiat money”; and (3) “establishment of arbitration on platform itself, which allows resolving main conflict situations without court involvement” [16]. These are some of the suggestions made to address the existing challenges. Generally, regulators need to work with the financial industry to make sure that all the benefits of DLTs are enjoyed while providing flexibility for the further innovation and development of this sector [141]. Yet, it is very difficult to strike this much needed balance at the international and national level.

This reality has been witnessed in the Qatari context where regulators are struggling to address the risks of DLTs technologies while reaping its benefits simultaneously. This led to a confusion for technology developers and investors, given that on the one hand the government banned trade in Bitcoin and cryptoasset services [134, 141], while still including Blockchain in its FinTech strategy. Given the nascent stage of the technology that emerged in 2008, its technical features and characteristics [100, 108], one would understand the Qatari confusion and cautious approach. In fact, the rest of the world are also struggling to regulate this technology, where few actors made a breakthrough. These include the EU, European countries, the USA and Canada, while legal developments are occurring at the international level via, for instance, the UN and other governmental and nongovernmental organizations [18, 60, 72, 107, 114, 121, 124, 128, 141]. Therefore, one should understand the Qatari situation and help government authorities, regulators and the various agencies to adopt rules that strike the needed balance.

The authors in this article provide recommendations to the various Qatari stakeholders involved in the regulation of DLTs. The authors suggest the use of three legal tools simultaneously through a multi-layered governance approach to the regulation of DLTs. These tools are: (1) embracing international regulations and standards; (2) replicating foreign regional and national rules that are appropriate and innovative; and (3) applying sandbox regulations to Blockchain products and services. The use of these legal tools together guarantees that Qatar benefits from the legal developments occurring at the international and national level concerning this field while simultaneously providing the necessary controlled environment at the domestic level to experiment with new DLT products and services. The authors believe that this approach is the most appropriate one to take given the existing challenges and the many uncertainties surrounding this technology, which may push a country to avoid its use despite its benefits. With this, Qatari industry would be able to reap the benefits of DLT while addressing its risks and challenges.
